# Outstanding Reviewers for *Chemical Science* in 2022

**DOI:** 10.1039/d3sc90103c

**Published:** 2023-06-29

## Abstract

We would like to take this opportunity to highlight the Outstanding Reviewers for *Chemical Science* in 2022, as selected by the editorial team for their significant contribution to the journal.
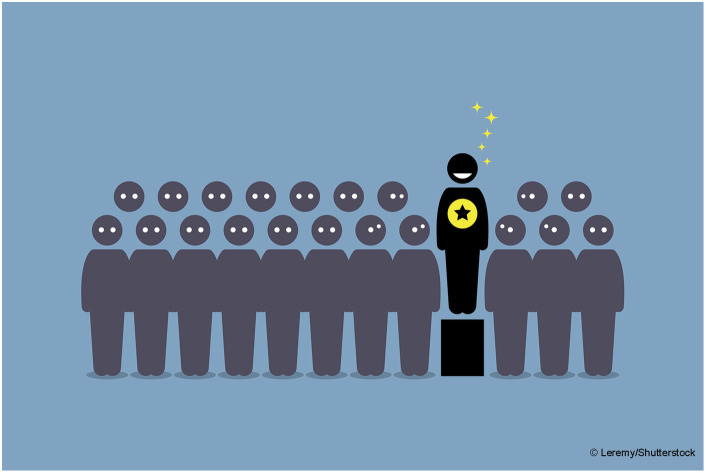

We would like to take this opportunity to thank all of *Chemical Science*'s reviewers for helping to preserve quality and integrity in chemical science literature.

We would also like to highlight the Outstanding Reviewers for *Chemical Science* in 2022. Each one of our outstanding peer reviewers has been carefully selected by our editorial team and includes active researchers who have made significant contributions to peer review and have gone above and beyond in their actions.

Our aim is to showcase and recognize the quality and diversity of the fantastic reviewers in the chemical sciences community. Therefore, the list below includes reviewers who have provided a higher-than-average number of top-quality reviewer reports, but we have also highlighted reviewers who have provided highly detailed reports, reviewers who have been specifically noted for providing constructive feedback that has helped authors improve their manuscripts, and also adjudicative reviewers who have provided thoughtful and robust reports for manuscripts with conflicting referee recommendations.

Alongside our list of Outstanding Reviewers for *Chemical Science*, we take pride in highlighting our fantastic reviewers all year long *via* our Reviewer Spotlight blog, which highlights a selection of reviewers every month. We continue to work on improving the diversity of our reviewer pool to reflect the diversity of the communities that we serve. You can read our Reviewer Spotlight blog at https://blogs.rsc.org/sc/category/reviewer-spotlight/ and can also keep an eye on our journal Twitter account @ChemicalScience to see which reviewers we will be promoting each month.

 

Our Outstanding Reviewers for *Chemical Science* in 2022 are:

 

Dr Anastassia Alexandrova

University of California, Los Angeles

ORCID: 0000-0002-3003-1911

 

Professor Rosana Álvarez Rodríguez

University of Vigo

ORCID: 0000-0001-5608-7561

 

Dr Christopher Barner-Kowollik

Queensland University of Technology

ORCID: 0000-0002-6745-0570

 

Dr Joaquin Barroso-Flores

Centro Conjunto de Investigación en Química Sustentable UAEM-UNAM

ORCID: 0000-0003-0554-7569

 

Dr Luca Bernardi

University of Bologna

ORCID: 0000-0002-7840-3200

 

Professor Ruth Brenk

University of Bergen

ORCID: 0000-0002-6204-5488

 

Dr Maria Contel

City University of New York

ORCID: 0000-0002-9825-4441

 

Dr Mark Crimmin

Imperial College London

ORCID: 0000-0002-9339-9182

 

Dr Stefanie Dehnen

Philipps-University Marburg

ORCID: 0000-0002-1325-9228

 

Dr William Evans

University of California Irvine

ORCID: 0000-0002-0651-418X

 

Professor Daniel Gryko

Polish Academy of Sciences

ORCID: 0000-0002-2146-1282

 

Dr Satoshi Horike

Kyoto University

ORCID: 0000-0001-8530-6364

 

Dr Ashlee Howarth

Concordia University

ORCID: 0000-0002-9180-4084

 

Professor Shina Caroline Lynn Kamerlin

Uppsala University

ORCID: 0000-0002-3190-1173

 

Dr Venkat Kapil

University of Cambridge

ORCID: 0000-0003-0324-2198

 

Dr Seda Keskin

Koç University

ORCID: 0000-0001-5968-0336

 

Dr Christoforos G. Kokotos

National and Kapodistrian University of Athens

ORCID: 0000-0002-4762-7682

 

Dr Søren Kramer

Technical University of Denmark

ORCID: 0000-0001-6075-9615

 

Dr Bhisma Kumar Patel

Indian Institute of Technology Guwahati

ORCID: 0000-0002-4170-8166

 

Professor Tatiana Martins

Federal University of Goiás

ORCID: 0000-0003-1209-9143

 

Dr Eric Masson

Ohio University

ORCID: 0000-0001-9387-4783

 

Dr Stefan Matile

University of Geneva

ORCID: 0000-0002-8537-8349

 

Professor Gloria Mazzone

University of Calabria

ORCID: 0000-0002-4686-6876

 

Professor David Mills

The University of Manchester

ORCID: 0000-0003-1575-7754

 

Dr Iwona Nierengarten

University of Strasbourg

ORCID: 0000-0003-0501-6768

 

Dr Wade Petersen

University of Cape Town

ORCID: 0000-0003-3215-5560

 

Dr Pachaiyappan Rajamalli

Indian Institute of Science

ORCID: 0000-0001-8079-0425

 

Dr Jacquelyne Read

Dartmouth College

ORCID: 0000-0002-0681-7703

 

Dr Neelanjana Sengupta

Indian Institute of Science Education and Research

ORCID: 0000-0003-0854-3467

 

Dr Xiadong Michael Shi

University of South Florida

ORCID: 0000-0002-3189-1315

 

Dr Douglas Stephan

University of Toronto

ORCID: 0000-0001-8140-8355

 

Dr Larissa von Krbek

University of Bonn (Rheinische Friedrich-Wilhelms-Universität Bonn)

ORCID: 0000-0002-4278-5235

 

Dr Joanna Wencel-Delord

University of Strasbourg

ORCID: 0000-0002-7094-5443

 

Dr Miho Yamauchi

Kyushu University

ORCID: 0000-0003-4752-172X

 

Dr Ting Yang

Northeastern University

ORCID: 0000-0001-5517-5658

 

Dr Ru-Jia Yu

Nanjing University

ORCID: 0000-0002-5376-3648

 

Professor Quichun Zhang

City University of Hong Kong

ORCID: 0000-0003-1854-8659

 

Dr Wei Zhang

University of Colorado Boulder

ORCID: 0000-0002-5491-1155

 

We would finally like to thank the *Chemical Science* Editorial Board and Advisory Board and the chemical sciences community for their continued support of the journal, as authors, reviewers and readers.

 

May Copsey, Executive Editor

## Supplementary Material

